# Sustained Long-Term Decline in Anti-HCV Neutralizing Antibodies in HIV/HCV-Coinfected Patients Five Years after HCV Therapy: A Retrospective Study

**DOI:** 10.3390/ph17091152

**Published:** 2024-08-30

**Authors:** Daniel Sepúlveda-Crespo, Camilla Volpi, Rafael Amigot-Sánchez, María Belén Yélamos, Cristina Díez, Julián Gómez, Víctor Hontañón, Juan Berenguer, Juan González-García, Rubén Martín-Escolano, Salvador Resino, Isidoro Martínez

**Affiliations:** 1 Unidad de Infección Viral e Inmunidad, Centro Nacional de Microbiología, Instituto de Salud Carlos III, Carretera Majadahonda-Pozuelo, Km 2.2, 28220 Majadahonda (Madrid), Spain; danisecre@hotmail.com (D.S.-C.); camilla.volpi1@studenti.unimi.it (C.V.); rafael.amigot@isciii.es (R.A.-S.); r.martin@isciii.es (R.M.-E.); 2 Centro de Investigación Biomédica en Red en Enfermedades Infecciosas (CIBERINFEC), Instituto de Salud Carlos III, Av. Monforte de Lemos, 3-5, 28029 Madrid, Spain; crispu82@gmail.com (C.D.); jbb4@me.com (J.B.); juangonzalezgar@gmail.com (J.G.-G.); 3 Dipartimento di Scienze Farmacologiche e Biomolecolari, Università degli Studi di Milano, Via Giuseppe Balzaretti, 9, 20133 Milan, Italy; 4 Departamento de Bioquímica y Biología Molecular, Facultad de Ciencias Químicas, Universidad Complutense, Pl de las Ciencias, 2, 28040 Madrid, Spain; mbyelamos@quim.ucm.es (M.B.Y.); jgomezgu@ucm.es (J.G.); 5 Unidad de Enfermedades Infecciosas/VIH, Hospital General Universitario Gregorio Marañón, C del Dr. Esquerdo, 46, 28007 Madrid, Spain; 6 Instituto de Investigación Sanitaria del Gregorio Marañón, C del Dr. Esquerdo, 46, 28007 Madrid, Spain; 7 Unidad de VIH, Servicio de Medicina Interna, Hospital Universitario La Paz, Paseo de la Castellana, 261, 28046 Madrid, Spain; victor.hontanon@gmail.com; 8 Instituto de Investigación Hospital Universitario La Paz, Paseo de la Castellana, 261, 28046 Madrid, Spain

**Keywords:** HIV, hepatitis C, anti-HCV therapy, broad-spectrum neutralizing antibodies, HIV/HCV coinfection, sustained virologic response

## Abstract

**Background:** This study evaluated titers and amplitudes of anti-E2 antibodies (anti-E2-Abs) and neutralizing antibodies against hepatitis C virus (HCV; anti-HCV-nAbs) in HIV/HCV-coinfected individuals over five years after successful HCV treatment completion. **Methods:** We retrospectively analyzed 76 HIV/HCV-coinfected patients achieving sustained virologic response post-HCV treatment. Plasma levels of anti-E2-Abs and anti-HCV-nAbs against five HCV genotypes (Gt1a, Gt1b, Gt2a, Gt3a, and Gt4a) were determined using ELISA and microneutralization assays, respectively. Statistical analyses comparing the three follow-up time points (baseline, one year, and five years post-HCV treatment) were performed using generalized linear mixed models, adjusting *p*-values with the false discovery rate (*q*-value). **Results:** Compared to baseline, anti-E2-Abs titers decreased at one year (1.9- to 2.3-fold, *q*-value < 0.001) and five years (3.4- to 9.1-fold, *q*-value < 0.001) post-HCV treatment. Anti-HCV-nAbs decreased 2.9- to 8.4-fold (*q*-value < 0.002) at one year and 17.8- to 90.4-fold (*q*-value < 0.001) at five years post-HCV treatment. Anti-HCV-nAbs titers against Gt3a were consistently the lowest. Nonresponse rates for anti-E2-Abs remained low throughout the follow-up, while anti-HCV-nAbs nonresponse rates increased 1.8- to 13.5-fold (*q*-value < 0.05) at five years post-HCV treatment, with Gt3a showing the highest nonresponse rate. **Conclusions:** Humoral immune responses against HCV decreased consistently one and five years post-HCV treatment, regardless of HCV genotype and previous HCV therapy or type of treatment (IFN- or DAA-based therapy). This decline was more pronounced for anti-HCV-nAbs, particularly against Gt3.

## 1. Introduction

The World Health Organization (WHO) estimates that approximately 58 million people globally have hepatitis C virus (HCV) infection, leading to approximately 290,000 deaths annually from liver-related problems [1,2]. Over 70% of HCV-infected patients reside in low- and middle-income countries, with 80% being unaware of their infection. Among those who are aware of their condition, only 5% receive treatment [1]. Previously, hepatitis C was treated with PEGylated interferon alpha (PEG-IFNα) and ribavirin (RBV), but this therapy had limited efficacy and was associated with severe side effects. Introducing oral direct-acting antivirals (DAAs), specifically IFN-free therapy, has transformed HCV care. This advancement ensures a sustained virologic response (SVR) of over 95% and contributes to controlling the hepatitis C epidemic in countries with access to DAA therapy [3]. The WHO recommends treating all patients with chronic HCV infection with pan-genotypic DAAs, which achieve an SVR in over 85% of patients across all six major HCV genotypes. For treatment-naïve patients, the WHO suggests two primary regimens: glecaprevir/pibrentasvir and sofosbuvir/velpatasvir or daclatasvir [4].

However, despite the success of treatment, HCV reinfection remains a common occurrence after clearance [5], especially among individuals who persist with risky practices (injected drugs and risky sexual practices), such as men who have sex with men, in human immunodeficiency virus (HIV) pre-exposure prophylaxis programs [6,7,8]. This susceptibility to HCV reinfection may be attributed to a less effective protective immune response in these individuals [9].

Cross-reactive neutralizing antibodies against HCV (anti-HCV-nAbs) target mainly the E2 glycoprotein and are associated with the control and clearance of HCV infection, as well as with protection from HCV reinfection [10,11]. However, anti-E2 antibodies (anti-E2-Abs) and anti-HCV-nAbs exhibit a rapid decay in HIV/HCV-coinfected individuals after achieving SVR [12,13]. Therefore, inducing these anti-HCV-nAbs is vital for developing an effective prophylactic HCV vaccine. However, the unique features of the E2 glycoprotein challenge this goal [14].

## 2. Objective

This study aimed to evaluate the titers and the amplitude of anti-E2-Abs and anti-HCV-nAbs in HIV/HCV-coinfected individuals over five years after the successful completion of HCV treatment.

## 3. Results

### 3.1. Patient Characteristics

Table 1 summarizes the baseline characteristics of 76 HIV/HCV-coinfected individuals who met the study’s eligibility criteria. The median age was 51 years, with a male predominance (81.6%). High alcohol intake was reported by 47.3%, and 76.3% had a history of injection drug use. Regarding immune status, 32.4% had a nadir of <200 CD4^+^/mm^3^, while 52.6% had a baseline CD4^+^/mm^3^ > 500.

With regard to liver disease, 51.3% had a liver stiffness measurement (LSM) of ≥20 kPa, and 10.5% had decompensated cirrhosis. Besides, 56.6% had undergone previous HCV therapy, with 65.8% receiving IFN-based therapy at baseline. Concerning HCV viral markers, HCV Gt1 (75.0%) was the most prevalent in the study population, followed by Gt3 (13.2%) and Gt4 (9.2%), and 62.7% had an HCV-RNA viral load ≥ 850,000 IU/mL. Additional details on individual patients’ HCV genotypes and antiviral treatment histories are provided in Supplementary Table S1.

### 3.2. Anti-HCV Antibody Titers

**Figure 1:**
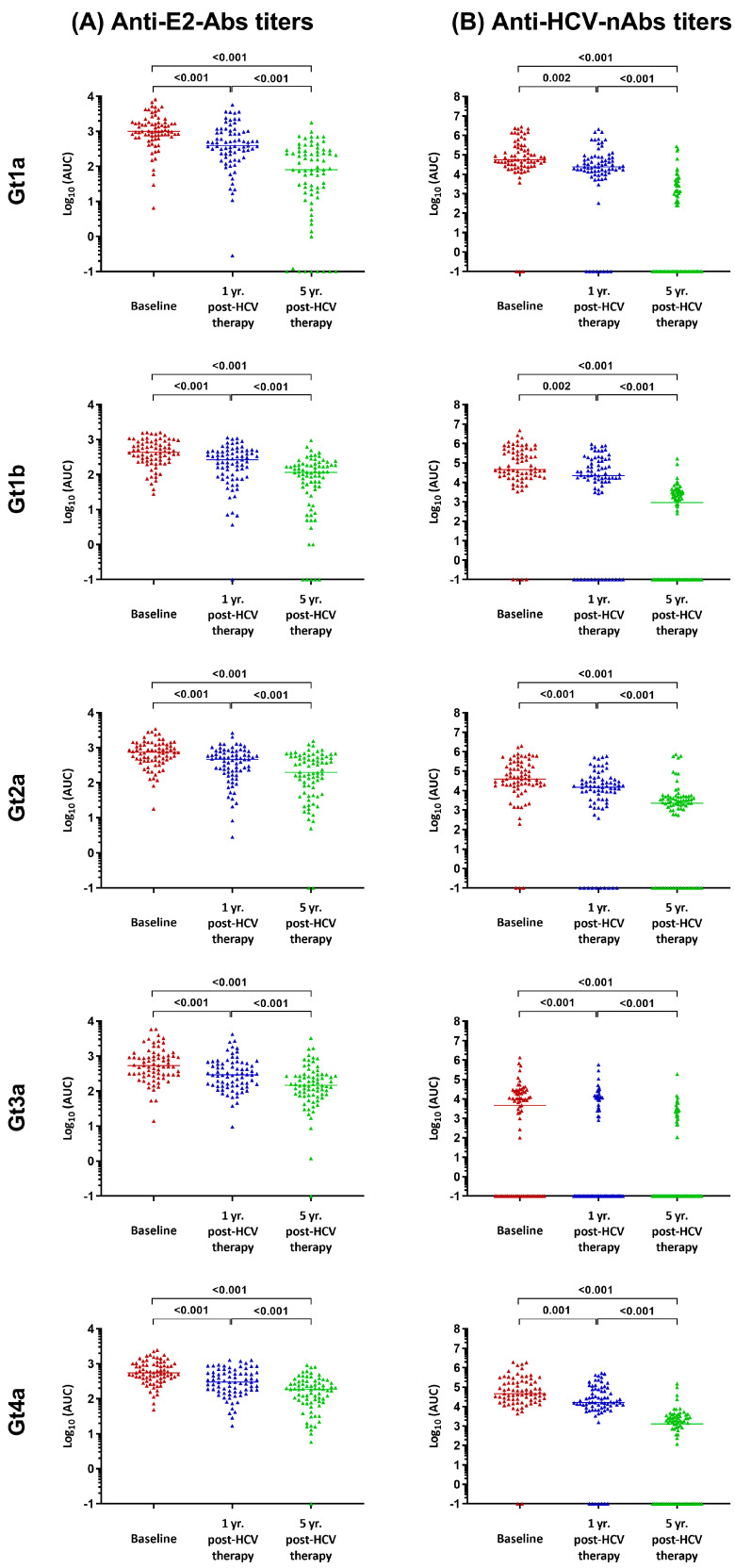
Anti-E2 antibodies ((**A**), anti-E2-Abs) and neutralizing antibodies against HCV ((**B**), anti-HCV-nAbs) titers during follow-up across HCV genotypes. Statistics: The data include individual values from each patient and the corresponding medians. Significant differences were determined using GLMM with gamma distribution and link (log). *p*-values were adjusted by the FDR (*q*-value). Abbreviations: AUC = area under the curve (arbitrary units); FDR = false discovery rate; GLMM = generalized linear mixed model; Gt = HCV genotype; HCV = hepatitis C virus; Yr = year.

At baseline, all patients exhibited the highest plasma anti-E2-Abs and anti-HCV-nAbs titers for each of the tested HCV genotypes (Figure 1). However, these titers were significantly reduced one year and five years post-HCV treatment (*q*-value < 0.05; Figure 1 and Supplementary Table S2). Plasma anti-E2-Abs titers significantly decreased between 1.9- and 2.3-fold at one year post-HCV treatment (*q*-value < 0.001), and a more substantial decrease from 3.4- to 9.1-fold was observed at five years post-HCV treatment (*q*-value < 0.001; Supplementary Table S2).

Concurrently, plasma anti-HCV-nAbs titers showed a significant decrease (Figure 1), ranging from 2.9- to 8.4-fold at one year post-HCV treatment (*q*-value ≤ 0.002). Notably, these reductions were more pronounced at five years post-HCV treatment, ranging from 17.8- to 90.4-fold (*q*-value < 0.001; Supplementary Table S2). Overall, anti-HCV-nAbs titers against Gt3a consistently remained the lowest at the three study time points, in comparison to other chimeric HCV viruses, ranging from 2.8- to 12.9-fold (*q*-value < 0.05; Supplementary Table S3).

### 3.3. Anti-HCV Nonresponse Rate

**Figure 2:**
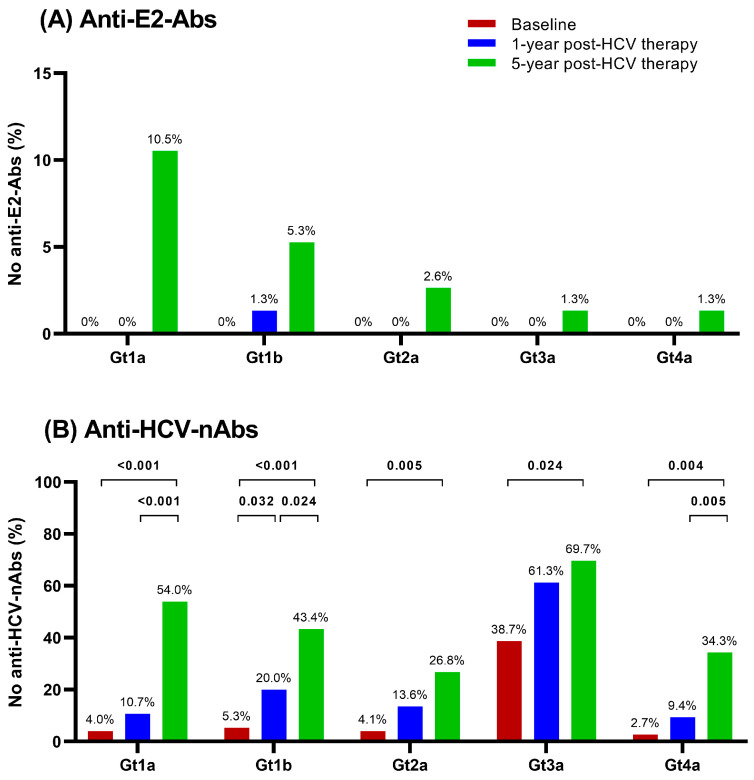
Nonresponse rate of anti-E2 antibodies ((**A**), anti-E2-Abs) and neutralizing antibodies against HCV ((**B**), anti-HCV-nAbs) during follow-up across HCV genotypes. Statistics: Significant differences were determined using GLMM with binomial distribution and link (logit). *p*-values were adjusted by the FDR (*q*-value). Abbreviations: FDR = false discovery rate; GLMM = generalized linear mixed model; Gt = HCV genotype; HCV = hepatitis C virus.

All patients showed a consistently low nonresponse rate to anti-E2-Abs throughout the follow-up period (Figure 2A). At baseline, anti-E2-Abs against the five HCV genotypes (Gt1a, Gt1b, Gt2a, Gt3a, and Gt4a) were detected in all patients. During follow-up, there was an increase in the nonresponse rate for anti-E2-Abs at five years post-HCV treatment, particularly noticeable for Gt1a (10.5%) and Gt1b (5.3%), but these changes did not attain statistical significance after the false discovery rate (FDR) adjustment.

We also studied anti-HCV-nAbs nonresponse rates during the follow-up period (Figure 2B and Supplementary Table S4). At baseline, plasma anti-HCV-nAb levels were undetected in 2.7–5.3% of patients. At one year post-HCV treatment, there were no significant increases in the nonresponse rate for anti-HCV-nAbs, except for Gt1b (Figure 2B). In contrast, at five years post-HCV treatment, significant increases were observed against the five chimeric HCV viruses (*q*-value ≤ 0.05; Figure 2B), with a 1.8- to 13.5-fold higher probability of having anti-HCV-nAbs nonresponse (*q*-value ≤ 0.05; Supplementary Table S4). Overall, anti-HCV-nAbs nonresponse rates against Gt3a consistently remained the highest at the three study time points compared to other chimeric HCV viruses, ranging from 1.3 to 14.5 times more likely to have a lack of anti-HCV-nAbs nonresponse (*q*-value < 0.05; Supplementary Table S5).

## 4. Discussion

This study explored the temporal trends in plasma titers of anti-HCV antibodies (anti-E2-Abs and anti-HCV-nAbs) over five years after successful anti-HCV therapy in HIV/HCV-coinfected patients. We found a substantial decline in anti-E2-Abs and anti-HCV-nAbs titers one year post-treatment. This decline was more pronounced five years post-HCV treatment and was particularly evident against Gt3. 

HCV exhibits significant genetic diversity, with 8 genotypes and 90 subtypes [15]. The envelope glycoproteins, E1 and E2, are primary targets for anti-HCV-nAbs. The E1 protein exhibits lower immunogenicity compared to E2, likely due to the limited availability of complete E1 structures and the fact that E2 largely occludes E1 in E1–E2 heterodimers [16,17]. In fact, only two main immunogenic regions in E1 that induce anti-HCV-nAbs have been identified, compared to the numerous well-characterized E2-derived anti-HCV-nAbs [18]. One of these E1-derived nAbs displays weak neutralizing activity [18], and they might not directly translate to protection from reinfection. However, the E2 protein is the main target for anti-HCV-nAbs because it is more accessible on the viral surface compared to E1 [14] and displays the highest amino acid variations among the HCV proteins [19]. Therefore, our study focused on assessing the neutralization capacity of plasma samples against chimeric HCV viruses expressing E2 glycoproteins from five distinct HCV genotypes. 

Following HCV infection, most antibodies target the HCV surface glycoproteins E1 and E2 of the virus. Anti-E2-Abs specifically recognize the E2 protein of the HCV. However, detecting anti-E2-Abs does not always indicate the ability to control or eliminate the infection, as not all anti-E2-Abs are neutralizing. Anti-HCV-nAbs, including some anti-E2 antibodies, can block the virus’s entry into liver cells, thereby preventing its replication. Therefore, analyzing anti-HCV-nAbs is crucial due to their critical role in HCV protection and clearance [20,21]. A delayed and weak anti-HCV-nAbs response contributes to chronic infection, while a prompt and robust response facilitates rapid HCV clearance after infection [10,22].

The microneutralization assay used in this study assesses the neutralizing capacity of antibodies against different HCV genotypes in cultured cells. This assay is crucial for evaluating the humoral immune response to HCV, determining the duration of long-term immune protection, and identifying patients at risk of relapse. This method complements PCR and sequencing, techniques designed to detect and characterize the virus. These methods, together, provide a more comprehensive view of the infection and the induced immune response.

Our study found that most patients exhibited neutralizing antibody responses against all HCV genotypes tested, regardless of their infecting genotype. This broad neutralization suggests a robust immune response to chronic HCV infection, which is typically asymptomatic for several years following initial exposure, delaying diagnosis until liver damage occurs. Individuals, particularly those in high-risk groups, may acquire multiple HCV infections with different genotypes over time. However, a single genotype usually dominates within a host at any given time. This may account for the absence of a diverse HCV genotype pool at initial assessment. Repeated exposure to various genotypes could stimulate the production of cross-reactive anti-HCV antibodies [23,24]. Moreover, the conserved nature of certain E2 epitopes likely contributes to the immune system’s ability to recognize and neutralize a variety of viral genotypes [25]. This is supported by previous studies demonstrating the presence of cross-reactive antibodies capable of neutralizing diverse HCV genotypes [12,13,26,27]. However, the neutralization titers against Gt3a were lower than those against other genotypes, particularly at five years post-HCV treatment, consistent with previous observations [12,13,26,27]. Neutralizing breadth in this study was assessed using a widely adopted cell-culture-derived infectious HCV (HCV_cc_) panel [28,29]. The lower titers of anti-HCV-nAbs against HCV Gt3a may be attributed to strain-specific rather than genotype-specific effects. Notably, HCV Gt3a (S52/JFH1) is particularly challenging to neutralize, a phenomenon not observed for other HCV Gt3a strains [29]. Due to the limited availability of recombinant HCV Gt3a strains in our laboratory, further investigations were constrained. On the other hand, previous studies have linked HCV Gt3a to higher rates of hepatic steatosis and increased risk of cirrhosis progression [30,31,32,33]. However, the mechanisms underlying the increased virulence of HCV Gt3a and the factors that may impair the immune system’s ability post-antiviral therapy remain elusive. Moreover, while ELISA and microneutralization assays are well established and validated, their sensitivity can influence results, particularly in long-term follow-up.

Earlier studies conducted by our group on HIV/HCV-coinfected patients found decreased anti-HCV-nAbs one year post-HCV treatment, without considering whether IFNα-based regimens or DAAs were employed [12,13]. Moreover, in one of these studies, we showed that HIV infection, even with suppressive cART, led to lower anti-E2 and HCV-nAbs titers compared to HCV-mono-infected individuals [12]. Our current study offers valuable additional insights, which are important for future vaccine development efforts, as it used data from real-world clinical settings, strengthening the generalizability of the findings beyond a controlled research setting. First, this study extended the follow-up period to five years, providing a more comprehensive picture of long-term anti-HCV antibody dynamics compared to our previous one-year analysis. Second, this study considered previous HCV therapy or type of treatment (IFN- or DAA-based therapy). Third, we also explored factors influencing reinfection risk by correlating antibody decline with specific patient characteristics. Our extended research revealed an even more pronounced reduction in anti-E2-Abs and anti-HCV-nAbs titers over five years post-HCV treatment. 

Interestingly, our analysis of HCV therapy approaches and clinical factors on anti-HCV antibody dynamics revealed no significant correlations between anti-E2-Ab or anti-HCV-nAb titers and previous HCV therapy, type of treatment (IFN- or DAA-based), age, HCV viral load, CD4+ T-cell count, CD4/CD8 ratio, or liver disease stages. While the immunosuppressive effect of IFN [34,35,36] or immune system modulation by DAA [37] might have been expected to influence antibody responses, our findings suggested otherwise. It is important to note that future studies with larger sample sizes might provide more definitive insights into these potential correlations.

The observed decline in anti-HCV antibodies aligns with other studies on HIV-infected patients treated for acute [38] or chronic [39] hepatitis C, suggesting that HCV elimination diminishes anti-HCV antibodies. The reduction of anti-HCV antibodies after SVR could be attributed to the loss of antigenic stimulation required for a lasting antibody response [12,13]. In this sense, it is important to remember the importance of viral antigens for B-cell stimulation and proliferation [40]. Furthermore, HIV/HCV-coinfection accelerates hepatic decompensation and severe liver events [41], impacting the humoral response against HCV [42]. HIV disrupts immunoglobulin class switching [43], leading to reduced high-affinity, broad-spectrum anti-HCV antibodies [44]. Similar studies on HIV coinfection with other pathogens, such as measles virus or *Streptococcus pneumoniae*, showed diminished pathogen-specific antibody production compared to individuals without HIV infection [45,46].

Reduced levels of anti-HCV antibodies may facilitate HCV reinfection. Therefore, monitoring anti-HCV antibody dynamics is crucial for assessing and mitigating the risk of HCV reinfection and for future vaccine development [47,48]. Further research is necessary to understand the impact of advanced cirrhosis on the long-term persistence or decline of anti-HCV antibodies post-treatment in HIV/HCV-coinfected patients.

This study also evaluated the correlation between anti-HCV antibody decline and liver disease severity by recruiting patients with a diverse range of liver disease stages. We employed transient elastography (FibroScan^®^, Echosens, Paris, France) to assess LSM using established cutoffs [49]. However, generalizing our findings requires considering two factors. First, our study population skewed toward participants with advanced fibrosis (LSM ≥ 20 kPa) and hepatic decompensation. Second, the absence of data on ascites, particularly for patients with decompensated cirrhosis, limited the accuracy of LSM in this subgroup, as ascites can interfere with the technique [50]. To address these limitations, future research should explore antibody dynamics in a larger cohort of HIV/HCV-coinfected patients, encompassing the full spectrum of liver disease severity and including data on the presence or absence of ascites.

### Limitations of This Study

Our study was subject to several limitations. First, the relatively small sample size limited the statistical power to detect significant differences between the groups. However, the inclusion of a repeated-measures design significantly improved the analytical capacity and allowed for within-subject comparisons, mitigating this limitation to some extent. Second, the absence of a comparative group of HCV-mono-infected patients precluded direct comparisons with HIV/HCV-coinfected patients that could have allowed a broader understanding of the immune dynamics in these patient populations. Third, the limited availability of clinical strains of HCV chimeric viruses containing HCV structural proteins (E2, E1, and core) restricted our ability to assess the neutralization capacity against viruses expressing E2 glycoproteins from additional HCV genotypes and to explore a broader range of neutralization responses, including those targeting E1 and core antigens. This could provide a more comprehensive understanding of the humoral immune response following HCV treatment in HIV/HCV-coinfected individuals. Fourth, the lack of data on specific E2 memory B lymphocytes in the blood prevented a deeper understanding of the dynamic changes in the HCV-specific humoral immune response over time. Finally, in the context of long-term follow-up, the results were not validated using other methods or kits. However, the assays used in this study are highly sensitive.

## 5. Materials and Methods

### 5.1. Design and Patients

This retrospective study utilized data and clinical samples from HIV/HCV-coinfected patients receiving anti-HCV therapy from 2000 to 2015, previously enrolled in two prospective observational studies conducted in Spain [51,52].

The study included individuals meeting the following criteria: (1) a confirmed chronic HCV infection and HIV infection; (2) HCV therapy with either IFN-based therapy (PEG-IFNα/RBV or PEG-IFNα/RBV/DAA) or oral DAA therapy; (3) achievement of SVR (undetectable HCV-RNA load 12–24 weeks—depending on regimen—after completing anti-HCV treatment); (4) availability of frozen plasma samples at three time points: baseline (start of HCV treatment), after about one year, and five years after successful HCV therapy completion; (5) a baseline CD4^+^ count ≥ 200 cells/µL; (6) stable combination antiretroviral therapy (cART) for over six months with an undetectable plasma HIV viral load (<50 copies/mL) or no need for cART, according to the guidelines used during the study period. Exclusion criteria included hepatitis B virus (HBV) coinfection or acute HCV infection at baseline, or patients who experienced HCV reinfection during follow-up. We also excluded patients taking immunosuppressive medications at baseline or follow-up due to the potential impact of these medications on antibody levels.

This study received approval from the Ethics Committee of the Instituto de Salud Carlos III (CEI PI 23_2011 and CEI PI 41_2014) and adhered to the ethical principles outlined in the 1975 Declaration of Helsinki. All patients provided written informed consent before participating in the study and joining the cohorts.

### 5.2. Clinical Data and Samples

We leveraged the databases from the two prospective observational studies to obtain epidemiological and clinical data. Peripheral blood samples were obtained via venipuncture and collected in ethylenediaminetetraacetic-acid-containing tubes. On the same day of collection, these samples were promptly sent to the HIV HGM BioBank, where they underwent processing and were subsequently stored at −80 °C until required for further analysis.

### 5.3. Laboratory Assays

#### 5.3.1. Cell Culture

Human-hepatoma-derived Huh7.5 cells were obtained from Apath LLC (Brooklyn, NY, USA), and the Huh7.5.1 clone 2 was generously provided by Dr. Francis V. Chisari (The Scripps Research Institute, La Jolla, CA, USA). Both cell lines were cultured in Dulbecco’s Modified Eagle Medium (DMEM; Lonza, Basel, Switzerland) supplemented with 10% fetal bovine serum (FBS; Biological Industries, Beit Haemek, Israel), 4 mM L-glutamine (Lonza), and an antibiotic cocktail (100 U/mL penicillin and 100 U/mL streptomycin; Lonza) at 37 °C in a 5% CO_2_ atmosphere. The cells were split every 2 to 3 days to maintain their growth and viability.

#### 5.3.2. Cloning, Expression, and Purification of E2 Glycoproteins

The DNA sequences encoding the ectodomain of the E2 glycoprotein (residues 384–661; E2661) from various HCV genotypes, including Gt1a (H77; GenBank accession no. EU363761), 1b (J4; FJ230881), 2a (JFH1; AB047639), 3a (S52; EU204645), and 4a (ED43; EU363760), were modified to incorporate a six-histidine tag (His tag) at the 5’ end. Subsequently, these sequences were inserted into a baculovirus transfer vector, pAcGP67A (Pharmingen, San Diego, CA, USA), using a method previously described [53], with some modifications, as detailed in previous work conducted by our laboratory [12,13].

#### 5.3.3. Chimeric Viruses

The plasmid encoding the JFH1 genome (Gt2a) was obtained from Apath LLC [54]. Dr. Jens Bukh (Copenhagen University Hospital, Copenhagen, Denmark) generously provided the plasmids encoding JFH1-based chimeric viruses, which contained the 3′ and 5′ ends and the NS3-NS5B region of JFH1 (essential for replication and production of viral particles), as well as the core-NS2 region from selected genotypes, including Gt1a (H77/JFH1), Gt1b (J4/JFH1), Gt3a (S52/JFH1), and Gt4a (ED43/JFH1) [26,55,56]. HCV_cc_ was produced from plasmid-transcribed RNAs using a method previously described [12,13].

#### 5.3.4. Quantitative ELISA Antibody Titration and HCV Neutralization Assays

As previously detailed in our published works [12,13], we quantified the anti-E2-Abs levels against several recombinant HCV-E2 glycoproteins (Gt1a, Gt1b, Gt2a, Gt3a, and Gt4a) using an enzyme-linked immunosorbent assay (ELISA). We also evaluated the antibodies’ ability to neutralize different HCV genotypes (anti-HCV-nAbs), including JFH1 Gt2a and JFH1-based recombinant Gt1a (H77), Gt1b (J4), Gt3a (S52), and Gt4a (ED43) chimeric HCV viruses. These specific HCV genotypes were selected based on their alignment with the infecting HCV genotypes observed in the study patients. Patient plasma was analyzed at three distinct time points: baseline, around one year after HCV therapy, and around five years post-HCV therapy. Non-linear regression one-phase decay curves were fitted, and the area under the curve for both assays was performed using GraphPad Prism v9.0 (GraphPad Software, Inc., San Diego, CA, USA). This study used a pool of plasma samples confirmed negative for anti-HCV antibodies using the Murex anti-HCV v4.0 ELISA (DiaSorin Diagnostics, Dartford, UK). The cutoff value for distinguishing positive from negative results in the ELISA was established based on the mean absorbance of the negative control wells plus three standard deviations [57].

### 5.4. Outcomes

We analyzed two outcome variables during the follow-up: (i) plasma anti-HCV antibody titers (anti-E2-Abs or anti-HCV-nAbs) and (ii) the anti-HCV nonresponse rate, which represents the percentage of patients without detectable plasma anti-HCV antibodies (anti-E2-Abs or anti-HCV-nAbs).

### 5.5. Statistical Analysis

All statistical analyses were conducted using the Statistical Package for the Social Sciences (SPSS) 25.0 (IBM Corp., Chicago, IL, USA) and Stata 15.0 (StataCorp, College Station, TX, USA). Plots and graphs were created using GraphPad Prism v9.0. 

Qualitative variables were presented as frequency and percentage, while quantitative variables were expressed as median values with interquartile range (IQR). 

Differences between the three follow-up time points (baseline, one year, and five years post-HCV treatment) were evaluated using generalized linear mixed models (GLMMs) with gamma distribution and link (log) for anti-HCV antibody titers. In contrast, GLMMs with Poisson distribution and link (log) were used for the anti-HCV nonresponse rate. These tests provided the arithmetic mean ratio (AMR), odds ratio (OR), and 95% confidence interval (95% CI). Pairwise comparisons between groups were performed using the *pwcompare* command.

The significance level was defined as *p* < 0.05 (two-tailed). The *p*-values were adjusted by the FDR (*q*-value) using the Benjamini and Hochberg procedure to exclude spurious associations.

## 6. Conclusions

Humoral immune responses against HCV exhibited a consistent decline over time, evident at one and five years post-HCV treatment, irrespective of the HCV genotype and previous HCV therapy or type of treatment (IFN- or DAA-based therapy). This decline was more pronounced in the case of anti-HCV-nAbs, particularly against Gt3.

## Figures and Tables

**Table 1:** Summary of baseline epidemiological and clinical characteristics of HIV/HCV-coinfected patients who underwent hepatitis C treatment.

Characteristics	Data
** No.**	76
** Epidemiological data**	
** Age (years), median [IQR]**	51 [47–54]
** Gender (male), *n* (%)**	62 (81.6)
** BMI (kg/m^2^), median [IQR]**	24.7 [21.7–28.7]
** Smoker, *n* (%)**	
- Never	5 (6.6)
- Previous (>6 months)	21 (27.6)
- Current	50 (65.8)
** Alcohol intake (>50 g/day), *n* (%)**	
- Never	40 (52.6)
- Previous (>6 months)	33 (43.4)
- Current	3 (3.9)
** Intravenous drug user, *n* (%)**	
- Never	18 (23.7)
- Previous (>6 months)	58 (76.3)
- Current	0 (0)
**HIV markers**	
** Prior AIDS, *n* (%)**	3 (3.9)
** ** **Nadir CD4^+^/mm^3^, median [IQR]**	145.0 [95.0–238.5]
** Nadir < 200 CD4^+^/mm^3^, *n* (%)**	24 (32.4)
** Baseline CD4^+^/mm^3^, median [IQR]**	518.0 [294.0–721.8]
** Baseline > 500 CD4^+^/mm^3^, *n* (%)**	40 (52.6)
**HIV antiretroviral therapy, *n* (%)**	
** NRTI + NNRTI**	19 (27.5)
** NRTI + II**	30 (43.5)
** NRTI + PI**	15 (21.7)
** PI+II + NNRTI/MVC**	2 (2.9)
** Others**	3 (4.3)
**Liver disease markers**	
** LSM (kPa), median [IQR]**	20.3 [12.4–34.8]
- 9.5 to 12.4 kPa	19 (25.0)
- 12.5 kPa to 19.9 kPa	18 (23.7)
- ≥20 kPa	39 (51.3)
** Hepatic decompensation, *n* (%)**	8 (10.5)
**HCV therapy** **, *n* (%)**	
** Previous HCV therapy**	43 (56.6)
** Baseline HCV Therapy**	
- IFN-based therapy	50 (65.8)
- IFN-free DAAs therapy	26 (34.2)
**HCV markers**	
** HCV genotype, *n* (%)**	
- 1	57 (75.0)
- 3	10 (13.2)
- 4	7 (9.2)
- Others/Unknown	2 (2.6)
** ** **Log_10_ HCV-RNA (IU/mL), median [IQR]**	6.1 [5.8–6.6]
** HCV-RNA ≥ 850,000 IU/mL, *n* (%)**	47 (62.7)

Statistics: The values are expressed as the absolute number (percentage) and median [interquartile range]. Abbreviations: AIDS = acquired immunodeficiency syndrome; BMI = body mass index; HCV = hepatitis C virus; HCV-RNA = HCV plasma viral load; HIV = human immunodeficiency virus; IFNα = interferon-alpha; II = integrase inhibitor; IP = protease inhibitor; IQR = interquartile range; LSM = liver stiffness measure; MVC = maraviroc; NNRTI = non-nucleoside reverse transcriptase inhibitor; NRTI = nucleoside reverse transcriptase inhibitor.

## Data Availability

Data is contained within the article and Supplementary Materials. The datasets generated during the current study are available from the corresponding author on reasonable request.

## Supplementary Materials

The following supporting information can be downloaded at: https://www.mdpi.com/article/10.3390/ph17091152/s1, Table S1: HCV infecting genotype and antiviral therapies for HIV and HCV in study patients; Table S2: Comparison of anti-E2 antibody titers (anti-E2-Abs) and neutralizing antibody titers against HCV (anti-HCV-nAbs) between baseline, one-year post-HCV therapy, and five-year post-HCV therapy; Table S3: Comparison of neutralizing antibody titers (anti-HCV-nAbs) against Gt3 in relation to those of other chimeric HCV viruses during the follow-up period; Table S4: Comparison of the nonresponse rates of neutralizing antibodies against HCV (anti-HCV-nAbs) between baseline, one-year post-HCV therapy, and five-year post-HCV therapy; Table S5: Comparison of the nonresponse rates of neutralizing antibodies (anti-HCV-nAbs) against Gt3 in relation to those of other chimeric HCV viruses during the follow-up period.

## List of Abbreviations

AMR	arithmetic mean ratio
Anti-E2-Abs	anti-E2 antibodies
Anti-HCV-nAbs	neutralizing antibodies against hepatitis C virus
cART	combination antiretroviral therapy
DAA	direct-acting antiviral
DMEM	Dulbecco’s Modified Eagle Medium
ELISA	enzyme-linked immunosorbent assay
FBS	fetal bovine serum
FDR	false discovery rate
GLMM	generalized linear mixed model
Gt	genotype
HBV	hepatitis B virus
HCV	hepatitis C virus
HCV_cc_	cell-culture-derived infectious HCV
HIV	human immunodeficiency virus
IFN	interferon
IQR	interquartile range
LSM	liver stiffness measurement
OR	odds ratio
PEG-IFNα	pegylated interferon alpha
RBV	ribavirin
RNA	ribonucleic acid
SVR	sustained virologic response
WHO	World Health Organization

## Appendix A

The GESIDA 3603b Cohort Study Group

Hospital General Universitario Gregorio Marañón, Madrid: A Carrero, P Miralles, JC López, F Parras, B Padilla, T Aldamiz-Echevarría, F Tejerina, C Díez, L Pérez-Latorre, C Fanciulli, I Gutiérrez, M Ramírez, S Carretero, JM Bellón, J Bermejo, and J Berenguer.

Hospital Universitario La Paz, Madrid: V Hontañón, JR Arribas, ML Montes, I Bernardino, JF Pascual, F Zamora, JM Peña, F Arnalich, M Díaz, and J González-García.

Hospital de la Santa Creu i Sant Pau, Barcelona: P Domingo and JM Guardiola.

Hospital Universitari Vall d’Hebron, Barcelona: E Van den Eynde, M Pérez, E Ribera, and M Crespo.

Hospital Universitario Ramón y Cajal, Madrid: JL Casado, F Dronda, A Moreno, MJ Pérez-Elías, MA Sanfrutos, S Moreno, and C Quereda.

Hospital Universitario Príncipe de Asturias, Alcalá de Henares: A Arranz, E Casas, J de Miguel, S Schroeder, and J Sanz.

Hospital Universitario de La Princesa, Madrid: J Sanz and I Santos.

Hospital Donostia, San Sebastián: MJ Bustinduy, JA Iribarren, F Rodríguez-Arrondo, and MA Von-Wichmann.

Hospital Clínico San Carlos, Madrid: J Vergas and MJ Téllez.

Hospital Universitario San Cecilio, Granada: D. Vinuesa, L. Muñoz, and J. Hernández-Quero.

Hospital Clínico Universitario, Valencia: A Ferrer and MJ Galindo.

Hospital General Universitario, Valencia: L Ortiz and E Ortega.

Hospital Universitari La Fe, Valencia: M Montero, M Blanes, S Cuellar, J Lacruz, M Salavert, and J López-Aldeguer.

Hospital Universitario de Getafe, Getafe: G Pérez and G Gaspar.

Fundación SEIMC-GESIDA, Madrid: M Yllescas, P Crespo, E Aznar, and H Esteban.

The ESCORIAL study group.

Hospital General Universitario Gregorio Marañón (Madrid, Spain): Cristina Díez, Luis Ibáñez, Leire Pérez-Latorre, Diego Rincón, Teresa Aldámiz-Echevarría, Vega Catalina, Pilar Miralles, Teresa Aldámiz-Echevarría, Francisco Tejerina, María C Gómez-Rico, Esther Alonso, José M Bellón, Rafael Bañares, and Juan Berenguer.

Hospital Universitario La Paz/IdiPAZ (Madrid, Spain): José Arribas, José I Bernardino, Ana Delgado, Carmen Busca, Javier García-Samaniego, Víctor Hontañón, Luz Martín-Carbonero, Rafael Micán, María L Montes-Ramírez, Victoria Moreno, Antonio Olveira, Ignacio Pérez-Valero, Eulalia valencia, and Juan González-García.

Hospital Universitario Puerta de Hierro (Madrid, Spain): Elba Llop and José Luis Calleja.

Hospital Universitario Ramón y Cajal (Madrid, Spain): Javier Martínez and Agustín Albillos.

Fundación SEIMC/GeSIDA (Madrid, Spain): Marta de Miguel, María Yllescas, and Herminia Esteban.
