# Supplementary material for: Sustained Long-Term Decline in Anti-HCV Neutralizing Antibodies in HIV/HCV-Coinfected Patients Five Years after HCV Therapy: A Retrospective Study
Source: Pharmaceuticals (Basel). 2024 Aug 30;17(9):1152. doi: 10.3390/ph17091152 (PMC11434851; doi:10.3390/ph17091152)
Supplement: Supplementary file 1 [file pharmaceuticals-17-01152-s001.zip › pharmaceuticals-3162313-supplementary.pdf]

Table S1. HCV infecting genotype and antiviral therapies for HIV and HCV in study patients.

Patient (#)	HCV genotype	HCV therapy	HIV antiretroviral therapy
**1**	3	SOF + DCV	2 NRTI + II
**2**	1b	SOF + SMV	2 NRTI + II
**3**	1a	SOF + DCV	2 NRTI + II
**4**	4	SOF + LDV	
**5**	1a	SOF + DCV	PI + NNRTI + II
**6**	1b	SOF + LDV	PI + II + MVC
**7**	4	SOF + LDV	NRTI + PI
**8**	1b	SOF + LDV	NNRTI + II
**9**	1b	SOF + LDV	2 NRTI + II
**10**	1a	3 DAAs (unspecified)	2 NRTI + II
**11**	4	SOF + DCV	
**12**	1a	SOF + SMV	2 NRTI + II
**13**	1a	SOF + SMV	2 NRTI + II
**14**	1a	SOF + DCV	2 NRTI + PI
**15**	1a	SOF + DCV	2 NRTI + II
**16**	1a	SOF + SMV	2 NRTI + II
**17**	4	SOF + LDV	2 NRTI + II
**18**	4	SOF + LDV	2 NRTI + NNRTI
**19**	1a	SOF + LDV	2 NRTI + II
**20**	1a	SOF + SMV	2 NRTI + II
**21**	1a	SOF + LDV	NNRTI + II
**22**	1a	SOF + LDV	2 NRTI + NNRTI
**23**	4	SOF + LDV	2 NRTI + II
**24**	1a	SOF + DCV + SMV	
**25**	3	SOF + DCV	2 NRTI + PI
**26**	1a	SOF + LDV	2 NRTI + NNRTI
**27**	1b	PEG-IFNα + RBV + PI	2 NRTI + NNRTI
**28**	1a	PEG-IFNα + RBV + PI	
**29**	1a	PEG-IFNα + RBV + PI	
**30**	1a	PEG-IFNα + RBV + PI	2 NRTI + PI
**31**	1a	PEG-IFNα + RBV + PI	2 NRTI + PI
**32**	1b	PEG-IFNα + RBV + PI	2 NRTI + PI
**33**	1b	PEG-IFNα + RBV + PI	2 NRTI + PI
**34**	1a	PEG-IFNα + RBV + PI	2 NRTI + II

**35**	1b	PEG-IFNα + RBV + PI	2 NRTI + NNRTI
**36**	3	PEG-IFNα + RBV	2 NRTI + PI
**37**	3	PEG-IFNα + RBV	2 NRTI + NNRTI
**38**	1a	PEG-IFNα + RBV + PI	2 NRTI + II
**39**	1b	PEG-IFNα + RBV + PI	2 NRTI + II
**40**	MG	PEG-IFNα + RBV + PI	2NRTI + PI
**41**	1a	PEG-IFNα + RBV + PI	2 NRTI + II
**42**	1a	PEG-IFNα + RBV	2 NRTI + NNRTI
**43**	1a	PEG-IFNα + RBV + PI	2 NRTI + II
**44**	3	PEG-IFNα + RBV	2 NRTI + PI
**45**	1a	PEG-IFNα + RBV + PI	2 NRTI + NNRTI
**46**	1b	PEG-IFNα + RBV + PI	II + PI
**47**	IG	PEG-IFNα + RBV + PI	NRTI + PI
**48**	1b	PEG-IFNα + RBV + PI	2 NRTI + PI
**49**	1a	PEG-IFNα + RBV	2 NRTI + II
**50**	IG	PEG-IFNα + RBV + PI	2 NRTI + II
**51**	3	PEG-IFNα + RBV + SOF	
**52**	IG	PEG-IFNα + RBV + SOF	
**53**	IG	PEG-IFNα + RBV + PI	2 NRTI + II
**54**	3	PEG-IFNα + RBV	2 NRTI + NNRTI
**55**	3	PEG-IFNα + RBV + SOF	2 NRTI + NNRTI
**56**	IG	PEG-IFNα + RBV + PI	2 NRTI + II
**57**	1a	PEG-IFNα + RBV + PI	2 NRTI + II
**58**	1a	PEG-IFNα + RBV + PI	2 NRTI + II
**59**	1a	PEG-IFNα + RBV + PI	2 NRTI + NNRTI
**60**	1b	PEG-IFNα + RBV + PI	2 NRTI + NNRTI
**61**	1a	PEG-IFNα + RBV + PI	2 NRTI + NNRTI
**62**	1b	PEG-IFNα + RBV + PI	2 NRTI + PI
**63**	1a	PEG-IFNα + RBV	2 NRTI + II
**64**	1b	PEG-IFNα + RBV + PI	2 NRTI + II
**65**	1b	PEG-IFNα + RBV + PI	2 NRTI + II
**66**	MG	PEG-IFNα + RBV + PI	2 NRTI + NNRTI
**67**	1b	PEG-IFNα + RBV + PI	2 NRTI + NNRTI
**68**	3	PEG-IFNα + RBV	2 NRTI + NNRTI
**69**	4	PEG-IFNα + RBV + PI	2 NRTI + PI
**70**	1a	PEG-IFNα + RBV + PI	2 NRTI + II

**71**	1b	PEG-IFNα + RBV + PI	2 NRTI + NNRTI
**72**	1a	PEG-IFNα + RBV + PI	2 NRTI + PI
**73**	1b	PEG-IFNα + RBV + PI	2 NRTI + NNRTI
**74**	1a	PEG-IFNα + RBV + PI	2 NRTI + NNRTI
**75**	3	PEG-IFNα + RBV	NRTI + NNRTI + II
**76**	1a	PEG-IFNα + RBV + PI	2 NRTI + II

Abbreviations: Patients #**1-26** are HIV/HCV-coinfected individuals who underwent treatment with IFN-free DAA therapy. Patients #**27-76** are HIV/HCV-coinfected individuals who received IFN*α*-based therapy. DAA = direct-acting antiviral; DCV = daclatasvir; HCV = hepatitis C virus; HIV = human immunodeficiency virus; IFN*α* = interferon alpha; IG = indeterminate HCV genotypes; II = integrase inhibitor; LDV = ledipasvir; MG = mixed HCV genotypes; MVC = maraviroc; NNRTI = non-nucleoside reverse transcriptase inhibitor; NRTI = nucleoside reverse transcriptase inhibitor; Peg-IFN*α* = pegylated IFN*α*; PI = protease inhibitor; RBV = ribavirin; SMV = simeprevir; SOF = sofosbuvir.

Table S2. Comparison of anti-E2 antibody titers (anti-E2-Abs) and neutralizing antibody titers against HCV (anti-HCV-nAbs) between baseline, one-year post-HCV therapy, and five-year post-HCV therapy.

.	**Baseline *vs.* 1-year post-HCV therapy**			**Baseline *vs.* 5-year post-HCV therapy**		
	**AMR (95%CI)**	***p*-value**	***q*-value**	**AMR (95%CI)**	***p*-value**	***q*-value**
	**Anti-E2-Abs**					
Gt1a	2.3 (3.3; 1.6)	**<0.001**	**<0.001**	9.1 (13.2; 6.2)	**<0.001**	**<0.001**
Gt1b	2.1 (2.7; 1.6)	**<0.001**	**<0.001**	5 (6.5; 3.8)	**<0.001**	**<0.001**
Gt2a	2.1 (2.6; 1.7)	**<0.001**	**<0.001**	3.6 (4.5; 2.9)	**<0.001**	**<0.001**
Gt3a	1.9 (2.3; 1.6)	**<0.001**	**<0.001**	3.4 (4.1; 2.8)	**<0.001**	**<0.001**
Gt4a	1.9 (2.3; 1.6)	**<0.001**	**<0.001**	3.7 (4.4; 3.1)	**<0.001**	**<0.001**
	**Anti-HCV-nAbs**					
Gt1a	2.9 (5.7; 1.5)	**0.002**	**0.002**	90.4 (204.5; 40)	**<0.001**	**<0.001**
Gt1b	3 (6; 1.5)	**0.002**	**0.002**	84.5 (185.1; 38.6)	**<0.001**	**<0.001**
Gt2a	3.7 (7.1; 1.9)	**<0.001**	**<0.001**	17.8 (37.3; 8.5)	**<0.001**	**<0.001**
Gt3a	8.4 (20.9; 3.3)	**<0.001**	**<0.001**	34.9 (99.5; 12.3)	**<0.001**	**<0.001**
Gt4a	3 (5.5; 1.6)	**0.001**	**0.001**	49.5 (97.8; 25.1)	**<0.001**	**<0.001**

Statistics: Data were calculated using GLMM. *P*-values were adjusted by the FDR (*q*-value). Significant differences are shown in bold. **Abbreviations**: 95%CI = 95% confidence interval; AMR = arithmetic mean ratio; FDR = false discovery rate; GLMM = generalized linear mixed model; Gt = HCV genotype; HCV = hepatitis C virus.

Table S3. Comparison of neutralizing antibody titers (anti-HCV-nAbs) against Gt3 in relation to those of other chimeric HCV viruses during the follow-up period.

	AMR (95%CI)	*p*-value	*q*-value
**Baseline**			
**Gt1a**	8.8 (4.4; 17.6)	**<0.001**	**<0.001**
**Gt1b**	9.1 (4.5; 18.6)	**<0.001**	**<0.001**
**Gt2a**	4.8 (2.4; 9.6)	**<0.001**	**<0.001**
**Gt3a**	Ref.		
**Gt4a**	5.5 (2.7; 11.2)	**<0.001**	**<0.001**
**1-year post-HCV therapy**			
**Gt1a**	9.7 (4.3; 22.2)	**<0.001**	**<0.001**
**Gt1b**	8.4 (3.7; 19)	**<0.001**	**<0.001**
**Gt2a**	4.5 (2; 10.1)	**<0.001**	**<0.001**
**Gt3a**	Ref.		
**Gt4a**	5.2 (2.3; 11.9)	**<0.001**	**<0.001**
**5-year post-HCV therapy**			
**Gt1a**	2.8 (1; 7.8)	**0.051**	**0.051**
**Gt1b**	3.4 (1.3; 9.2)	**0.014**	**0.019**
**Gt2a**	12.9 (4.7; 35.5)	**<0.001**	**<0.001**
**Gt3a**	Ref.		
**Gt4a**	4 (1.5; 10.6)	**0.005**	**0.010**

Statistics: Data were calculated using GLMM. *P*-values were adjusted by the FDR (*q*-value). Significant differences are shown in bold. **Abbreviations**: 95%CI = 95% confidence interval; AMR = arithmetic mean ratio (Gt/Gt3); FDR = false discovery rate; GLMM = generalized linear mixed model; Gt = HCV genotype; HCV = hepatitis C virus.

Table S4. Comparison of the nonresponse rates of neutralizing antibodies against HCV (anti-HCV-nAbs) between baseline, one-year post-HCV therapy, and five-year post-HCV therapy.

	**Baseline *vs.* 1-year post-HCV therapy**			**Baseline *vs.* 5-year post-HCV therapy**		
	OR (95%CI)	***p*-value**	***q*-value**	**OR (95%CI)**	***p*-value**	***q*-value**
**Gt1a**	2.7 (0.7; 10.1)	0.147	0.147	13.5 (4.2; 43.6)	**<0.001**	**<0.001**
**Gt1b**	3.8 (1.2; 11.3)	**0.019**	0.095	8.2 (2.9; 23)	**<0.001**	**<0.001**
**Gt2a**	3.3 (0.9; 12.1)	0.067	0.112	6.6 (2; 22.2)	**0.002**	**0.003**
**Gt3a**	1.6 (1; 2.5)	0.052	0.112	1.8 (1.1; 2.8)	**0.011**	**0.011**
**Gt4a**	3.5 (0.7; 16.9)	0.118	0.147	12.8 (3; 54.1)	**0.001**	**0.002**

Statistics: Data were calculated using GLMM. *P*-values were adjusted by the FDR (*q*-value). Significant differences are shown in bold. **Abbreviations**: 95%CI = 95% confidence interval; OR = odds ratio; FDR = false discovery rate; GLMM = generalized linear mixed model; Gt = HCV genotype; HCV = hepatitis C virus.

Table S5. Comparison of the nonresponse rates of neutralizing antibodies (anti-HCV-nAbs) against Gt3 in relation to those of other chimeric HCV viruses during the follow-up period.

	OR (95%CI)	*p*-value	*q*-value
Baseline			
**Gt1a**	9.7 (31.7; 2.9)	**<0.001**	**<0.001**
**Gt1b**	7.3 (20.6; 2.5)	**<0.001**	**<0.001**
**Gt2a**	9.4 (30.7; 2.8)	**<0.001**	**<0.001**
**Gt3a**	Ref.		
**Gt4a**	14.5 (60.8; 3.5)	**<0.001**	**<0.001**
**1-year post-HCV therapy**			
**Gt1a**	5.8 (12.2; 2.7)	**<0.001**	**<0.001**
**Gt1b**	3.1 (5.5; 1.7)	**<0.001**	**<0.001**
**Gt2a**	4.6 (9.1; 2.3)	**<0.001**	**<0.001**
**Gt3a**	Ref.		
**Gt4a**	6.6 (14.6; 3)	**<0.001**	**<0.001**
**5-year post-HCV therapy**			
**Gt1a**	1.3 (1.9; 0.9)	**0.217**	**0.217**
**Gt1b**	1.6 (2.5; 1)	**0.033**	**0.044**
**Gt2a**	2.6 (4.4; 1.6)	**<0.001**	**<0.001**
**Gt3a**	Ref.		
**Gt4a**	2 (3.3; 1.3)	**0.003**	**0.006**

Statistics: Data were calculated using GLMM. *P*-values were adjusted by the FDR (*q*-value). Significant differences are shown in bold. **Abbreviations**: 95%CI = 95% confidence interval; OR = odds ratio; FDR = false discovery rate; GLMM = generalized linear mixed model; Gt = HCV genotype; HCV = hepatitis C virus.
